# Distinct clinical phenotypes in acute heart failure identified by unsupervised clustering: a nationwide registry-based study

**DOI:** 10.3389/fcvm.2026.1862519

**Published:** 2026-08-25

**Authors:** Bo Eun Park, Dong Heon Yang, Eung Ju Kim, Seong Woo Han, Seong-Mi Park, Hyung-Seop Kim, Ju-Hee Lee, Hyo-Suk Ahn, Mi-Seung Shin, Hyun-Jai Cho, Jung Rang Park, Jin-Ok Jeong, Junho Hyun, Jin Oh Choi, Byung-Su Yoo, Seok-Min Kang, Dong-Ju Choi

**Affiliations:** 1 Division of Cardiology, Department of Internal Medicine, School of Medicine, Kyungpook National University, Daegu, Republic of Korea; 2 Division of Cardiology, Department of Internal Medicine, Kyungpook National University Hospital, Daegu, Republic of Korea; 3 Division of Cardiology, Department of Internal Medicine, Korea University Guro Hospital, Seoul, Republic of Korea; 4 Division of Cardiology, Department of Internal Medicine, Dongtan Sacred Heart Hospital, Hallym University, Hwaseong, Republic of Korea; 5 Division of Cardiology, Department of Internal Medicine, Keimyung University Dongsan Hospital, Daegu, Republic of Korea; 6 Division of Cardiology, Department of Internal Medicine, Keimyung University School of Medicine, Daegu, Republic of Korea; 7 Division of Cardiology, Department of Internal Medicine, Chungbuk National University Hospital, Chungbuk National University College of Medicine, Cheongju, Republic of Korea; 8 Division of Cardiology, Department of Internal Medicine, Uijeongbu St. Mary's Hospital, College of Medicine, The Catholic University of Korea, Uijeongbu, Republic of Korea; 9 Division of Cardiology, Department of Internal Medicine, Gachon University College of Medicine, Incheon, Republic of Korea; 10 Division of Cardiology, Department of Internal Medicine, Seoul National University College of Medicine, Seoul, Republic of Korea; 11 Division of Cardiology, Department of Internal Medicine, Seoul National University Hospital, Seoul, Republic of Korea; 12 Division of Cardiology, Department of Internal Medicine, Gyeongsang National University Hospital, Gyeongsang National University College of Medicine, Jinju, Republic of Korea; 13 Division of Cardiology, Department of Internal Medicine, Chungnam National University School of Medicine, Daejeon, Republic of Korea; 14 Division of Cardiology, Department of Internal Medicine, Chungnam National University Hospital, Daejeon, Republic of Korea; 15 Division of Cardiology, Department of Internal Medicine, Asan Medical Center, Seoul, Republic of Korea; 16 Division of Cardiology, Department of Internal Medicine, University of Ulsan College of Medicine, Seoul, Republic of Korea; 17 Division of Cardiology, Department of Internal Medicine, Cardiovascular Institute, Samsung Medical Center, Seoul, Republic of Korea; 18 Division of Cardiology, Department of Internal Medicine, Sungkyunkwan University School of Medicine, Seoul, Republic of Korea; 19 Division of Cardiology, Department of Internal Medicine, Wonju Severance Christian Hospital, Yonsei University Wonju College of Medicine, Wonju, Republic of Korea; 20 Division of Cardiology, Department of Internal Medicine, Yonsei University College of Medicine, Seoul, Republic of Korea; 21 Division of Cardiology, Department of Internal Medicine, Severance Cardiovascular Hospital, Seoul, Republic of Korea; 22 Division of Cardiology, Department of Internal Medicine, Cardiovascular Center, Seoul National University Bundang Hospital, Seoul National University College of Medicine, Seongnam, Republic of Korea

**Keywords:** acute heart failure, clinical phenotypes, clustering, prognosis, registry, risk stratification

## Abstract

**Background:**

Acute heart failure (AHF) is a highly heterogeneous clinical syndrome, posing challenges for risk stratification and management. Phenotype-based classification using unsupervised clustering may provide insights beyond conventional approaches.

**Methods:**

We analyzed a nationwide, prospective, multicenter registry of patients hospitalized with AHF. Baseline demographic characteristics, clinical variables, laboratory findings, and echocardiographic parameters obtained at index hospitalization were used for unsupervised clustering. K-means clustering was applied, and the optimal number of clusters was determined based on internal validation metrics and clinical interpretability. Clinical characteristics, treatment patterns, and outcomes were compared across clusters. Cox proportional hazards models were used to evaluate the association between cluster membership and clinical outcomes.

**Results:**

A total of 7,351 patients were classified into five distinct clinical phenotypes. The clusters demonstrated marked differences in baseline characteristics, comorbidity burden, hemodynamic profiles, and cardiac function. Treatment patterns, including guideline-directed medical therapy and supportive interventions, varied significantly across clusters. During follow-up, all-cause mortality differed significantly among clusters (*p* < 0.001), with Clusters 2, 3, and 5 remaining independently associated with increased mortality compared with Cluster 1 after multivariable adjustment. Heart failure hospitalization also differed significantly across clusters (*p* < 0.001), although differences were less pronounced after multivariable adjustment. The composite endpoint of all-cause mortality or heart failure hospitalization showed clear separation across clusters (*p* < 0.001). In multivariable analysis, cluster membership remained independently associated with all-cause mortality and the composite endpoint.

**Conclusion:**

In this large, nationwide cohort of patients with AHF, unsupervised clustering identified five clinically distinct phenotypes with significantly different characteristics, management patterns, and outcomes. These findings support the clinical relevance of phenotype-based classification and highlight its potential to enhance risk stratification and inform more personalized management strategies in AHF.

## Introduction

Acute heart failure (AHF) is a leading cause of hospitalization worldwide and is associated with substantial morbidity and mortality despite advances in pharmacological and device-based therapies (1, 2). Patients presenting with AHF represent a highly heterogeneous population with respect to demographics, comorbid conditions, hemodynamic profiles, and underlying cardiac structure and function (3). This clinical heterogeneity poses a major challenge for risk stratification and therapeutic decision-making in routine practice (4, 5).

Conventional classifications of heart failure, such as those based on left ventricular ejection fraction or clinical congestion status, provide useful but limited insights into the complexity of AHF (6, 7). These approaches often fail to capture the multidimensional nature of patient presentations and may not adequately explain the wide variability in treatment response and outcomes observed in real-world settings (8–10).

Unsupervised clustering techniques offer a data-driven approach to identifying distinct patient phenotypes by simultaneously considering multiple clinical variables without prespecified assumptions (11, 12). Previous studies have suggested that phenotype-based classification using clustering methods may improve prognostic stratification in heart failure (13–15); however, evidence in large, contemporary cohorts of AHF patients remains limited, particularly in Asian populations (16–18).

Therefore, the aim of the present study was to identify distinct clinical phenotypes of AHF using an unsupervised clustering approach applied to a nationwide, prospective heart failure registry (18). We sought to characterize differences in baseline clinical features, in-hospital management and discharge therapies, and long-term clinical outcomes across identified clusters. We further evaluated whether cluster membership was independently associated with mortality and heart failure–related outcomes beyond established clinical risk factors.

By identifying distinct clinical phenotypes of acute heart failure through an unsupervised clustering approach, this study aims to provide a more comprehensive framework for understanding patient heterogeneity, clinical management patterns, and long-term prognosis (19, 20). Such phenotype-based stratification may offer insights beyond conventional classifications and support more individualized approaches to risk assessment in acute heart failure.

## Methods

### Study population and data source

The KorHF III registry is a prospective, multicenter cohort study conducted at 47 tertiary hospitals in Korea, enrolling patients hospitalized with acute heart failure (AHF) between March 2018 and December 2022 (18). Patients were included based on clinical signs or symptoms of heart failure, evidence of pulmonary congestion on chest radiography or physical examination, or structural or functional cardiac abnormalities identified by echocardiography (3, 16).

This study was based on a multicenter, prospective registry of patients hospitalized with acute heart failure (17). Patients were consecutively enrolled according to predefined diagnostic criteria. Demographic characteristics, clinical presentation, laboratory findings, echocardiographic parameters, in-hospital management, discharge medications, and longitudinal outcomes were prospectively collected using a standardized case report form.

All 7,351 eligible patients were included in the clustering analysis. Missing values in variables used for clustering were handled during data preprocessing as described below. All analyses were performed using de-identified data. The study protocol was approved by the institutional review board of each participating center, and informed consent was obtained from all patients or their legal representatives.

Details of the KorHF III registry have been described previously (17, 18).

### Variables used for clustering analysis

Unsupervised clustering was performed using baseline variables obtained at the time of index hospitalization. Variables were selected to represent multidimensional clinical characteristics routinely available in acute heart failure care, including demographic characteristics, hemodynamic parameters, laboratory findings, radiographic findings, echocardiographic measurements, and major comorbidities, as previously described in phenotype-based heart failure studies (6, 7, 12).

The following 17 variables were included in the clustering analysis: age, sex, systolic blood pressure at admission, heart rate at admission, hemoglobin, serum creatinine, serum sodium, log-transformed NT-proBNP, left ventricular ejection fraction measured by Simpson's method, pulmonary congestion on chest radiography, pleural effusion on chest radiography, cardiomegaly on chest radiography, hypertension, diabetes mellitus, chronic kidney disease, ischemic heart disease, and chronic obstructive pulmonary disease. Detailed definitions, coding, missing values, and preprocessing procedures for all variables are provided in Supplementary Table S1.

Missing values in clustering variables were handled using multiple imputation before clustering. Continuous variables were subsequently standardized to z-scores to ensure equal contribution of each variable to the clustering algorithm (20). Categorical variables were encoded as binary indicators after imputation.

Outcome variables were not included in the clustering process.

### Clustering methodology

An unsupervised clustering approach was applied to identify distinct phenotypic subgroups among patients hospitalized with acute heart failure using the k-means algorithm implemented in R statistical software (6, 11, 21).

Continuous variables were standardized to z-scores before clustering, whereas categorical variables were encoded as binary variables. The optimal number of clusters was determined using internal validation metrics, including within-cluster sum of squares (elbow method), together with clinical interpretability (21). Based on these criteria, a five-cluster solution (*K* = 5) was selected as the final model. The elbow method supported the selection of five clusters, and silhouette analysis was performed as an additional internal validation of the clustering solution (Supplementary Figures S1, S2).

To assess the robustness of the clustering solution, additional internal validation analyses were performed using silhouette analysis. Clinical consistency across phenotypes was qualitatively evaluated. Cluster labels were assigned after completion of clustering according to the dominant clinical characteristics observed within each cluster and were not used during the clustering process itself.

**Figure 1:**
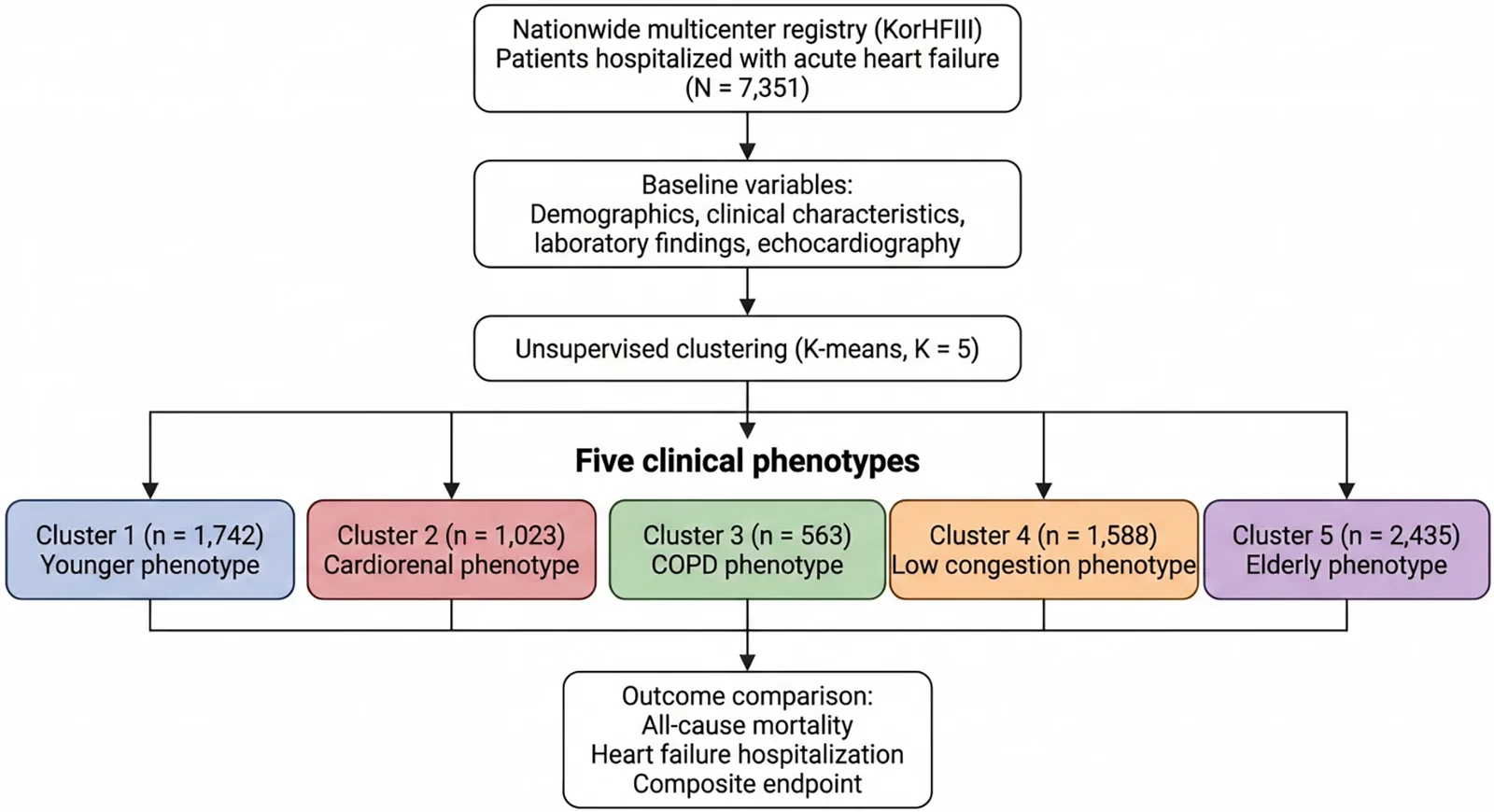
Study design and identification of clinical phenotypes of acute heart failure. Patients hospitalized with acute heart failure were identified from a nationwide, multicenter registry (KorHFIII). Baseline demographic characteristics, clinical variables, laboratory findings, and echocardiographic parameters collected at index hospitalization were used for unsupervised clustering analysis. Using K-means clustering (*K* = 5), patients were classified into five distinct clinical phenotypes, which were subsequently compared in terms of clinical characteristics, management strategies, and outcomes.

Each patient was assigned to a single cluster, which was subsequently used for descriptive, treatment pattern, and outcome analyses. The overall study design and clustering workflow are summarized in Figure 1.

### Clinical characteristics and treatment patterns

Baseline clinical characteristics were summarized and compared across clusters (Table 1). Discharge medications and in-hospital supportive therapies, including intensive care unit (ICU) admission, mechanical ventilation, inotropic support, and renal replacement therapy, were evaluated according to cluster classification (Table 2).

**Table 1:** Baseline clinical characteristics according to cluster classification.

Variable	Cluster 1 (*n* = 1,742)	Cluster 2 (*n* = 1,023)	Cluster 3 (*n* = 563)	Cluster 4 (*n* = 1,588)	Cluster 5 (*n* = 2,435)	*P* value
Age, years	53.9 ± 14.1	72.8 ± 12.1	76.3 ± 10.2	67.6 ± 12.1	78.0 ± 9.1	<0.001
Male sex, *n* (%)	1,269 (72.8)	573 (56.0)	351 (62.3)	982 (61.8)	996 (40.9)	<0.001
SBP at admission, mmHg	132.2 ± 28.6	141.5 ± 30.5	134.1 ± 27.1	124.4 ± 24.5	139.1 ± 28.8	<0.001
Heart rate, bpm	104.0 ± 26.2	87.4 ± 22.4	91.8 ± 25.3	79.2 ± 19.7	91.1 ± 23.8	<0.001
Hemoglobin, g/dL	14.4 ± 2.0	10.8 ± 2.1	12.2 ± 2.4	13.0 ± 2.1	11.3 ± 2.1	<0.001
Creatinine, mg/dL	1.1 ± 0.5	3.1 ± 2.5	1.3 ± 0.7	1.1 ± 0.5	1.2 ± 0.6	<0.001
Sodium, mmol/L	138.1 ± 3.6	137.6 ± 4.5	137.7 ± 4.7	138.6 ± 3.8	137.5 ± 4.9	<0.001
LVEF, %	26.8 ± 9.6	37.5 ± 12.6	37.8 ± 14.2	38.9 ± 13.1	40.2 ± 13.4	<0.001
Log-transformed NT-proBNP	8.3 (7.9–8.7)	8.7 (8.3–10.0)	8.3 (8.1–8.8)	7.4 (6.4–8.3)	8.3 (8.2–9.1)	<0.001
Pulmonary congestion, *n* (%)	676 (38.8)	522 (51.0)	305 (54.2)	155 (9.8)	1,357 (55.7)	<0.001
Pleural effusion, *n* (%)	992 (56.9)	682 (66.7)	346 (61.5)	186 (11.7)	1,858 (76.3)	<0.001
Cardiomegaly, *n* (%)	1,408 (80.8)	767 (75.0)	397 (70.5)	583 (36.7)	1,961 (80.5)	<0.001
Hypertension, *n* (%)	543 (31.2)	783 (76.5)	343 (60.9)	882 (55.5)	1,868 (76.7)	<0.001
Diabetes mellitus, *n* (%)	381 (21.9)	644 (63.0)	193 (34.3)	544 (34.3)	1,142 (46.9)	<0.001
Chronic kidney disease, *n* (%)	27 (1.5)	1,002 (97.9)	107 (19.0)	56 (3.5)	0 (0.0)	<0.001
Ischemic heart disease, *n* (%)	150 (8.6)	426 (41.6)	167 (29.7)	456 (28.7)	784 (32.2)	<0.001
COPD, *n* (%)	0 (0.0)	8 (0.8)	563 (100.0)	0 (0.0)	0 (0.0)	<0.001

Values are presented as mean ± SD, median (IQR), or *n* (%). NT-proBNP values were log-transformed.

**Table 2:** Discharge medications and supportive therapies according to cluster classification.

Variable	Cluster 1 (*n* = 1,742)	Cluster 2 (*n* = 1,023)	Cluster 3 (*n* = 563)	Cluster 4 (*n* = 1,588)	Cluster 5 (*n* = 2,435)	*P* value
ACE inhibitor	348 (20.0%)	63 (6.2%)	55 (9.8%)	180 (11.3%)	254 (10.4%)	<0.001
ARB	713 (40.9%)	420 (41.1%)	247 (43.9%)	617 (38.9%)	1,082 (44.4%)	<0.001
ARNI	384 (22.0%)	179 (17.5%)	86 (15.3%)	318 (20.0%)	344 (14.1%)	<0.001
Beta-blocker	1,345 (77.2%)	684 (66.9%)	327 (58.1%)	1,076 (67.8%)	1,535 (63.0%)	<0.001
MRA	1,263 (72.5%)	333 (32.6%)	338 (60.0%)	786 (49.5%)	1,375 (56.5%)	<0.001
Loop diuretics	1,386 (79.6%)	737 (72.0%)	467 (82.9%)	1,008 (63.5%)	1,943 (79.8%)	<0.001
SGLT2 inhibitor	357 (20.5%)	98 (9.6%)	65 (11.5%)	260 (16.4%)	365 (15.0%)	<0.001
ICU admission	526 (30.2%)	311 (30.4%)	132 (23.4%)	452 (28.5%)	621 (25.5%)	0.001
Mechanical ventilation	80 (4.6%)	52 (5.1%)	35 (6.2%)	63 (4.0%)	102 (4.2%)	0.217
Inotropic support	359 (20.6%)	181 (17.7%)	64 (11.4%)	274 (17.3%)	333 (13.7%)	<0.001
Renal replacement therapy	177 (10.2%)	531 (51.9%)	174 (30.9%)	473 (29.8%)	797 (32.7%)	<0.001

Values are presented as *n* (%).

ICU admission was defined as admission to an ICU at any time during the index hospitalization for acute heart failure and was analyzed as an indicator of disease severity and supportive care requirements.

### Outcomes

The primary outcome was all-cause mortality during follow-up. Secondary outcomes included heart failure hospitalization and a composite endpoint of all-cause death or heart failure hospitalization, defined as time to first event.

Time-to-event outcomes were calculated from the date of index hospitalization to the occurrence of each event or the last available follow-up.

For Kaplan–Meier analyses, follow-up was administratively truncated at 500 days because relatively few patients remained at risk thereafter. Cox proportional hazards models were also performed using follow-up restricted to the first 500 days. Descriptive outcome frequencies shown in Table 3 were based on the entire available follow-up period.

**Table 3:** Clinical outcomes according to cluster classification.

Outcome	Cluster 1 (*n* = 1,742)	Cluster 2 (*n* = 1,023)	Cluster 3 (*n* = 563)	Cluster 4 (*n* = 1,588)	Cluster 5 (*n* = 2,435)	*P* value
All-cause mortality, *n* (%)	172 (9.9%)	419 (41.0%)	222 (39.4%)	258 (16.2%)	829 (34.0%)	<0.001
HF hospitalization, *n* (%)	225 (12.9%)	310 (30.3%)	152 (27.0%)	272 (17.1%)	493 (20.2%)	<0.001
Composite outcome^a^, *n* (%)	352 (20.2%)	584 (57.1%)	300 (53.3%)	448 (28.2%)	1,096 (45.0%)	<0.001
Follow-up duration, days, median (IQR)	367 (325–397)	360 (194–404)	369 (299–416)	367 (324–399)	365 (213–406)	0.010

a Composite outcome was defined as all-cause mortality or heart failure hospitalization.

HF, heart failure; IQR, interquartile range.

### Statistical analysis

Continuous variables are presented as mean ± standard deviation or median (interquartile range), as appropriate, and categorical variables as counts and percentages. Group comparisons across clusters were performed using analysis of variance or the Kruskal–Wallis test for continuous variables and the chi-square or Fisher's exact test for categorical variables.

To evaluate potential patterns of missingness, baseline demographic and clinical characteristics were compared between patients with available and missing measurements of left ventricular ejection fraction and NT-proBNP in the original registry dataset before imputation. Continuous variables were compared using Welch's *t*-test, and categorical variables were compared using the chi-square test or Fisher's exact test, as appropriate. The results are presented in Supplementary Table S3.

Kaplan–Meier curves were constructed to estimate time-to-event outcomes according to cluster classification, and differences between clusters were assessed using the log-rank test. Cox proportional hazards regression models were used to evaluate the association between cluster membership and clinical outcomes, with results reported as hazard ratios and 95% confidence intervals. Multivariable models were adjusted for age, sex, systolic blood pressure at admission, renal function, left ventricular ejection fraction, and major comorbidities including hypertension, diabetes mellitus, chronic kidney disease, ischemic heart disease, and chronic obstructive pulmonary disease. The proportional hazards assumption was assessed and found to be acceptable.

All statistical tests were two-sided, and a *p* value < 0.05 was considered statistically significant. Statistical analyses were performed using R software (version 4.1.3).

As a sensitivity analysis, hierarchical clustering using Ward's minimum variance method (Ward.D2) was performed with the same standardized variables used for the primary k-means clustering. A five-cluster solution was generated for comparison with the primary clustering results, and concordance of the overall phenotypic structure was assessed qualitatively.

## Results

### Patient clustering and baseline characteristics

Using unsupervised clustering analysis based on baseline clinical, laboratory, and echocardiographic variables at the time of index hospitalization, patients with acute heart failure were classified into five distinct clusters. The clusters demonstrated clearly differentiated clinical profiles, including age distribution, comorbidity burden, hemodynamic status, renal function, and cardiac structure and function (Table 1).

Significant differences across clusters were observed for most baseline characteristics, indicating substantial heterogeneity in patient phenotypes at presentation. These differences supported the validity of the clustering approach in identifying clinically meaningful subgroups.

Baseline characteristics differed significantly across clusters (Table 1). The mean age varied markedly across clusters, with Cluster 1 representing the youngest group (53.9 ± 14.1 years), whereas Cluster 5 and Cluster 3 comprised older populations (78.0 ± 9.1 and 76.3 ± 10.2 years, respectively; *p* < 0.001).

Hemodynamic profiles at admission also differed. Cluster 1 showed the highest heart rate (104.0 ± 26.2 bpm), while Cluster 4 had the lowest (79.2 ± 19.7 bpm; *p* < 0.001). Systolic blood pressure was highest in Cluster 2 (141.5 ± 30.5 mmHg) and lowest in Cluster 4 (124.4 ± 24.5 mmHg; *p* < 0.001).

Laboratory findings demonstrated substantial heterogeneity across clusters. Cluster 2 exhibited markedly elevated serum creatinine levels (3.1 ± 2.5 mg/dL), indicating a high burden of renal dysfunction, whereas other clusters showed relatively preserved renal function (*p* < 0.001). Hemoglobin levels were lowest in Cluster 2 (10.8 ± 2.1 g/dL), consistent with a higher prevalence of anemia (*p* < 0.001).

Cardiac functional parameters also differed significantly. Left ventricular ejection fraction was lowest in Cluster 1 (26.8 ± 9.6%), whereas Cluster 5 showed relatively preserved systolic function (40.2 ± 13.4%; *p* < 0.001). NT-proBNP levels were highest in Cluster 2 (8.7 (8.3–10.0)), suggesting more severe hemodynamic stress (*p* < 0.001).

Radiographic findings revealed distinct congestion patterns. Pulmonary congestion and pleural effusion were more frequently observed in Cluster 5 (55.7% and 76.3%, respectively), while Cluster 4 showed markedly lower rates (9.8% and 11.7%; *p* < 0.001). Cardiomegaly was prevalent in Clusters 1 and 5 (80.8% and 80.5%, respectively).

Comorbidities also varied substantially. Hypertension and diabetes were most prevalent in Clusters 2 and 5 (HTN: 76.5% and 76.7%; DM: 63.0% and 46.9%, respectively; *p* < 0.001). Chronic kidney disease was predominantly observed in Cluster 2 (97.9%), whereas chronic obstructive pulmonary disease was uniquely concentrated in Cluster 3 (100.0%) (*p* < 0.001).

Missingness analyses showed modest but statistically significant differences in several baseline characteristics between patients with available and missing LVEF or NT-proBNP measurements (Supplementary Table S3).

### Discharge medications and in-hospital management

Treatment patterns differed significantly across clusters. The use of beta-blockers varied across groups (Cluster 1: 77.2%; Cluster 2: 66.9%; Cluster 3: 58.1%; Cluster 4: 67.8%; Cluster 5: 63.0%; *p* < 0.001).

According to the classification used in our dataset, renin–angiotensin system inhibitors were analyzed as ACE inhibitors/ARBs and angiotensin receptor–neprilysin inhibitors (ARNIs). The use of ACE inhibitors showed marked variation across clusters (Cluster 1: 20.0%; Cluster 2: 6.2%; Cluster 3: 9.8%; Cluster 4: 11.3%; Cluster 5: 10.4%; *p* < 0.001), while ARB use was relatively similar but still statistically different (Cluster 1: 40.9%; Cluster 2: 41.1%; Cluster 3: 43.9%; Cluster 4: 38.9%; Cluster 5: 44.4%; *p* < 0.001). The use of ARNIs also differed significantly (Cluster 1: 22.0%; Cluster 2: 17.5%; Cluster 3: 15.3%; Cluster 4: 20.0%; Cluster 5: 14.1%; *p* < 0.001).

Mineralocorticoid receptor antagonists were more frequently used in Cluster 1 (72.5%) and Cluster 3 (60.0%) compared with Cluster 2 (32.6%), with significant differences across clusters (*p* < 0.001). Loop diuretics were widely used across all clusters but showed variation in prescription rates (63.5%–82.9%, *p* < 0.001). SGLT2 inhibitors were more frequently prescribed in Cluster 1 (20.5%) compared with other clusters (9.6%–16.4%, *p* < 0.001).

Regarding supportive therapies, ICU admission rates differed across clusters (23.4%–30.4%, *p* = 0.001). Inotropic support was more frequently used in Cluster 1 (20.6%) compared with Cluster 3 (11.4%) and Cluster 5 (13.7%) (*p* < 0.001). Renal replacement therapy was markedly higher in Cluster 2 (51.9%) compared with other clusters (10.2%–32.7%, *p* < 0.001). Mechanical ventilation did not differ significantly across clusters (*p* = 0.217).

These differences in treatment patterns further support the clinical heterogeneity of the identified clusters.

### Clinical outcomes according to cluster

Median follow-up duration differed modestly across clusters, ranging from 360 to 369 days (*p* = 0.010) (Table 3).

**Figure 2:**
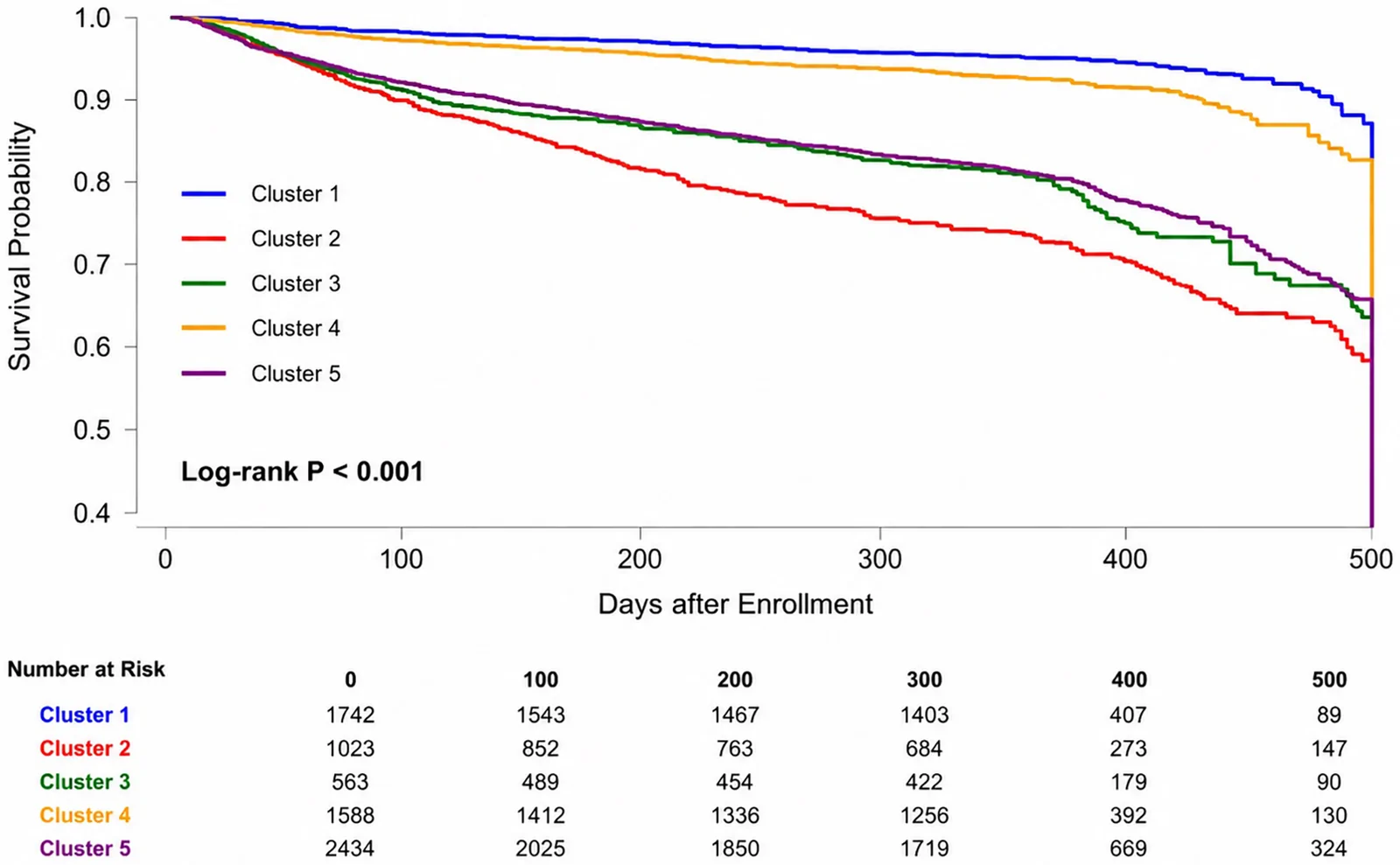
Kaplan–Meier curves for all-cause mortality according to clinical phenotypes of acute heart failure. Kaplan–Meier survival analysis of 500-day all-cause mortality demonstrated significant differences among the five clinical phenotypes identified by unsupervised clustering. The cumulative incidence of all-cause death within 500 days varied significantly across clusters (log-rank *p* < 0.001), with higher mortality observed in Cluster 2 and Cluster 3, and the lowest risk in Cluster 1.

During the entire available follow-up period, all-cause mortality differed significantly among clusters (Cluster 1: 9.9%; Cluster 2: 41.0%; Cluster 3: 39.4%; Cluster 4: 16.2%; Cluster 5: 34.0%; *p* < 0.001) (Table 3). When follow-up was truncated at 500 days, significant differences in all-cause mortality remained across clusters (log-rank *P* < 0.001) (Figure 2). Kaplan–Meier analysis demonstrated clear separation of survival curves across clusters, with the highest mortality observed in clusters characterized by advanced age, renal dysfunction, and hemodynamic instability.

**Figure 3:**
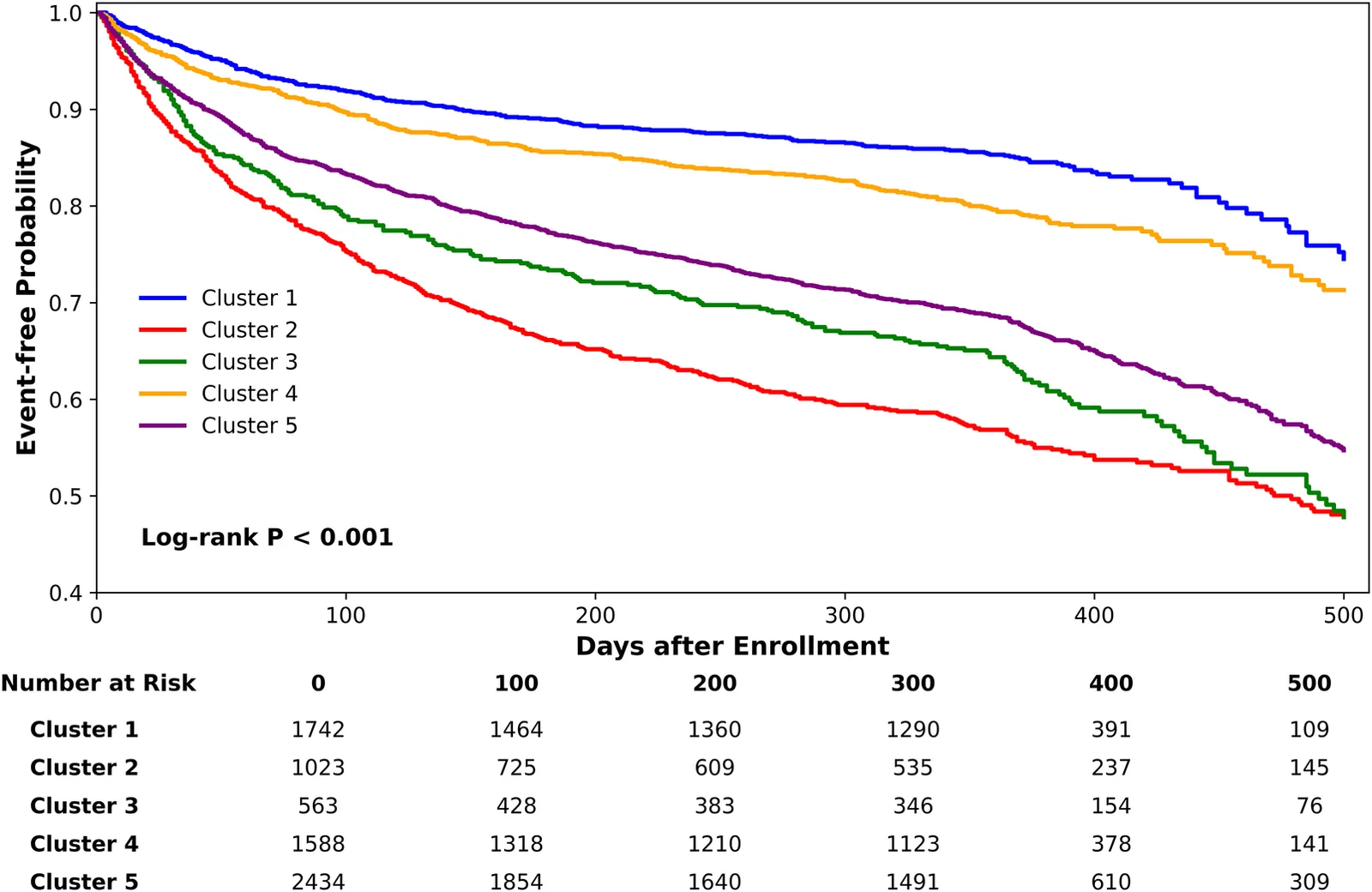
Kaplan–Meier curves for the composite endpoint of all-cause mortality or heart failure hospitalization according to clinical phenotypes of acute heart failure. Kaplan–Meier analysis of the 500-day composite endpoint demonstrated significant differences in the cumulative incidence of all-cause mortality or heart failure hospitalization among the five clinical phenotypes identified by unsupervised clustering (log-rank *p* < 0.001). Higher-risk clusters showed consistently increased event rates compared with the reference group.

During the entire available follow-up period, rates of heart failure hospitalization alone also differed across clusters (Cluster 1: 12.9%; Cluster 2: 30.3%; Cluster 3: 27.0%; Cluster 4: 17.1%; Cluster 5: 20.2%; *p* < 0.001) (Table 3). Differences in heart failure hospitalization within 500 days were also observed across clusters (log-rank *P* < 0.001) (Supplementary Figure S3). However, the magnitude of separation was less pronounced compared with mortality. During the entire available follow-up period, the composite endpoint of all-cause mortality or heart failure hospitalization differed significantly among clusters (Cluster 1: 20.2%; Cluster 2: 57.1%; Cluster 3: 53.3%; Cluster 4: 28.2%; Cluster 5: 45.0%; *p* < 0.001) (Table 3). Similar findings were observed for the composite endpoint when analyses were restricted to the first 500 days after enrollment (log-rank *P* < 0.001) (Figure 3). Kaplan–Meier curves for the composite endpoint demonstrated distinct risk trajectories across clusters.

### Cox proportional hazards analysis

In unadjusted Cox regression analyses restricted to the first 500 days after index hospitalization, cluster membership was significantly associated with all-cause mortality, heart failure hospitalization, and the composite endpoint (Table 4).

**Table 4:** Association between cluster membership and 500-day clinical outcomes.

Outcome	Comparison	Unadjusted HR (95% CI)	*P* value	Adjusted HR (95% CI)	*P* value
All-cause mortality	Cluster 2 vs. 1	4.54 (3.64–5.66)	<0.001	1.78 (1.36–2.34)	<0.001
	Cluster 3 vs. 1	3.77 (2.93–4.85)	<0.001	1.63 (1.23–2.17)	<0.001
	Cluster 4 vs. 1	1.49 (1.17–1.91)	0.002	1.15 (0.88–1.51)	0.292
	Cluster 5 vs. 1	3.51 (2.86–4.32)	<0.001	1.37 (1.07–1.76)	0.014
HF hospitalization	Cluster 2 vs. 1	2.75 (2.28–3.32)	<0.001	2.15 (1.67–2.76)	<0.001
	Cluster 3 vs. 1	2.29 (1.83–2.87)	<0.001	1.82 (1.41–2.36)	<0.001
	Cluster 4 vs. 1	1.31 (1.08–1.59)	0.007	1.35 (1.08–1.69)	0.008
	Cluster 5 vs. 1	1.72 (1.44–2.04)	<0.001	1.3 (1.04–1.63)	0.023
Composite (death or HF hospitalization)	Cluster 2 vs. 1	3.08 (2.67–3.56)	<0.001	1.86 (1.54–2.24)	<0.001
	Cluster 3 vs. 1	2.68 (2.27–3.17)	<0.001	1.71 (1.41–2.07)	<0.001
	Cluster 4 vs. 1	1.32 (1.14–1.54)	<0.001	1.22 (1.03–1.45)	0.025
	Cluster 5 vs. 1	2.18 (1.91–2.49)	<0.001	1.3 (1.10–1.53)	0.003

Adjusted models were adjusted for age, sex, systolic blood pressure at admission, hemoglobin, serum creatinine, serum sodium, left ventricular ejection fraction, and log-transformed NT-proBNP.

HR, hazard ratio; CI, confidence interval; HF, heart failure; NT-proBNP, N-terminal pro-B-type natriuretic peptide.

For all-cause mortality, Cluster 2 (adjusted HR 1.78, 95% CI 1.36–2.34, *p* < 0.001), Cluster 3 (adjusted HR 1.63, 95% CI 1.23–2.17, *p* < 0.001), and Cluster 5 (adjusted HR 1.37, 95% CI 1.07–1.76, *p* = 0.014) remained independently associated with increased mortality after multivariable adjustment, whereas Cluster 4 did not retain statistical significance.

For heart failure hospitalization, all clusters remained independently associated with increased risk after adjustment (Cluster 2: adjusted HR 2.15, 95% CI 1.67–2.76; Cluster 3: adjusted HR 1.82, 95% CI 1.41–2.36; Cluster 4: adjusted HR 1.35, 95% CI 1.08–1.69; Cluster 5: adjusted HR 1.30, 95% CI 1.04–1.63; all *p* < 0.05).

Similarly, for the composite endpoint, all clusters remained independently associated with higher risk compared with Cluster 1 after adjustment (Cluster 2: adjusted HR 1.86, 95% CI 1.54–2.24; Cluster 3: adjusted HR 1.71, 95% CI 1.41–2.07; Cluster 4: adjusted HR 1.22, 95% CI 1.03–1.45; Cluster 5: adjusted HR 1.30, 95% CI 1.10–1.53; all *p* < 0.05).

These findings indicate that the identified phenotypic clusters provide prognostic information beyond conventional clinical risk factors, even when analyses were restricted to the first 500 days of follow-up.

### Sensitivity analysis using Ward's hierarchical clustering

Sensitivity analysis using Ward's hierarchical clustering demonstrated partial concordance with the k-means-derived phenotypes. Although agreement was relatively strong for some clusters, other k-means clusters were distributed across multiple Ward-derived clusters. Therefore, this analysis should be interpreted as a sensitivity comparison supporting partial robustness, rather than as formal evidence of cluster stability. The cross-tabulation between the k-means-derived clusters and Ward-derived clusters is provided in Supplementary Table S2.

## Discussion

In this nationwide registry-based study, we identified five distinct clinical phenotypes of acute heart failure using an unsupervised clustering approach, each characterized by unique baseline profiles, management strategies, and clinical outcomes (16–18). These findings underscore the multidimensional heterogeneity of acute heart failure and demonstrate that data-driven phenotype classification can capture clinically meaningful differences not fully explained by conventional risk factors (3, 8). These findings highlight the limitations of conventional classification systems and emphasize the importance of integrating multidimensional clinical data to better capture patient heterogeneity in acute heart failure. Such an approach may facilitate more precise risk stratification and inform tailored therapeutic strategies in clinical practice.

### Comparison with previous phenotype-based studies

Previous studies have attempted to classify heart failure patients into distinct phenotypes using clustering or latent class analyses, primarily focusing on chronic heart failure populations or selected clinical trial cohorts (10, 11). These studies have demonstrated that phenotype-based classification can improve prognostic stratification beyond traditional measures such as left ventricular ejection fraction (12, 15). However, many prior analyses were limited by relatively small sample sizes, restricted variable sets, or lack of comprehensive outcome assessment.

In contrast, the present study extends existing evidence by applying an unsupervised clustering approach to a large, nationwide registry of patients hospitalized with acute heart failure. By incorporating routinely available demographic, laboratory, and echocardiographic variables, we identified clinically interpretable phenotypes that reflect real-world patient heterogeneity. Importantly, the identified clusters showed distinct management patterns and outcome trajectories, supporting the clinical relevance of this approach in an acute care setting.

### Interpretation of heart failure hospitalization patterns across clusters

In the present analysis, heart failure hospitalization alone differed significantly across clusters; however, the magnitude of separation was less pronounced compared with mortality. Several factors may explain this observation. First, hospitalization for heart failure is influenced by healthcare system factors, physician decision-making, and follow-up intensity, which may attenuate differences attributable solely to patient phenotype. Second, competing risk from early mortality in higher-risk clusters may reduce the observed incidence of subsequent hospitalization.

Notably, when mortality and heart failure hospitalization were analyzed as a composite endpoint, significant differences across clusters emerged. This finding suggests that composite outcomes may more comprehensively capture disease burden and clinical trajectory in acute heart failure than individual endpoints alone. Similar observations have been reported in prior heart failure studies, underscoring the limitations of hospitalization-based endpoints when interpreted in isolation (19, 21).

### Clinical implications and potential applications

The identification of distinct acute heart failure phenotypes has several important clinical implications. First, phenotype-based classification may enhance early risk stratification at the time of hospitalization by integrating multidimensional clinical information rather than relying on single parameters. Second, the observed differences in discharge medications and supportive therapies across clusters suggest that clinicians may already be intuitively tailoring management based on underlying patient profiles, albeit without a formalized framework.

From a practical standpoint, the clustering approach used in this study relies on variables that are routinely collected in clinical practice, facilitating potential translation into real-world decision support tools. Phenotype-guided strategies may help identify patients who require closer monitoring, intensified therapy, or early post-discharge follow-up (22). In clinical practice, these phenotypes could be automatically assigned using routinely available admission variables embedded within electronic health record systems. Such an approach could facilitate early identification of high-risk patients, support phenotype-specific monitoring and treatment strategies, and assist clinicians in prioritizing post-discharge follow-up according to individual risk profiles. Future studies incorporating machine learning–based models and external validation are warranted to refine these phenotypes and evaluate their role in guiding personalized therapeutic interventions.

Sensitivity analysis using Ward's hierarchical clustering demonstrated partial concordance with the k-means-derived phenotypes. Agreement was relatively strong for some clinically distinctive high-risk phenotypes, whereas other k-means clusters were distributed across multiple Ward-derived clusters. Therefore, this analysis should be interpreted as a sensitivity comparison providing partial support for the robustness of the identified phenotypes, rather than as formal evidence of cluster stability.

### Limitations

Several limitations of this study should be acknowledged. First, this was a *post hoc* analysis of a prospective registry, and although comprehensive clinical, laboratory, and echocardiographic data were available, residual confounding cannot be fully excluded. Second, the clustering approach was based on variables collected at the time of index hospitalization; therefore, changes in clinical status or treatment during follow-up were not incorporated into phenotype assignment. Third, although we performed elbow and silhouette analyses and compared the k-means solution with Ward's hierarchical clustering as a sensitivity analysis, we did not conduct a formal bootstrap- or resampling-based cluster-stability analysis, such as Jaccard similarity or adjusted Rand index. In addition, cluster reproducibility was not externally validated in an independent cohort. Therefore, the stability and generalizability of the identified phenotypes should be interpreted with caution, and these phenotypes should be considered hypothesis-generating rather than definitive classifications.

In addition, heart failure hospitalization may be influenced by healthcare system–related factors and competing risks, which may partly explain the relatively smaller separation observed in hospitalization outcomes across clusters compared with mortality. Post-discharge medical therapy may also have influenced subsequent heart failure hospitalization and was not incorporated into the multivariable models because the primary objective was to evaluate the prognostic significance of baseline phenotypes identified at index hospitalization. Finally, although the clustering methodology provides a data-driven framework for phenotype identification, causal inferences regarding treatment effects cannot be drawn from the present observational analysis.

## Conclusion

Overall, these findings support the clinical relevance of data-driven phenotyping in acute heart failure and highlight its potential to improve risk stratification and guide more personalized management strategies.

Using unsupervised clustering of routinely collected clinical data, we identified five distinct phenotypes of acute heart failure with significantly different clinical characteristics, management patterns, and outcomes. Cluster membership was independently associated with all-cause mortality and the composite endpoint of death or heart failure hospitalization.

These results further suggest that phenotype-based classification may improve risk stratification in acute heart failure and provide a foundation for more personalized therapeutic approaches. Further studies incorporating machine learning–based models and external validation are warranted to refine these phenotypes and extend their clinical applicability.

## Data Availability

The data analyzed in this study are derived from the KorHF III registry and contain sensitive patient information; therefore, the datasets are not publicly available, but reasonable requests for access to the data may be considered by the corresponding author, subject to approval by the Institutional Review Board of Kyungpook National University Hospital and compliance with relevant ethical and data protection regulations.

## Supplementary material

SUPPLEMENTARY FIGURE 1 Elbow plot for selection of the optimal number of clusters. The total within-cluster sum of squares decreased as the number of clusters increased, with an apparent inflection around K = 5. Based on the elbow method together with clinical interpretability, a five-cluster solution was selected for the final analysis.

SUPPLEMENTARY FIGURE 2 Silhouette analysis for k-means clustering. Average silhouette widths were calculated across different cluster solutions. A five-cluster solution was selected based on internal validation metrics together with clinical interpretability.

SUPPLEMENTARY FIGURE 3 Kaplan-Meier curves for 500-day heart failure hospitalization according to clinical phenotypes of acute heart failure. Kaplan–Meier analysis of 500-day heart failure hospitalization demonstrated significant differences among the five clinical phenotypes (log-rank p < 0.001), although the magnitude of separation was less pronounced than that observed for all-cause mortality and the composite endpoint.
