# Supplementary material for: Distinct clinical phenotypes in acute heart failure identified by unsupervised clustering: a nationwide registry-based study
Source: Front Cardiovasc Med. 2026 Aug 25;13:1862519. doi: 10.3389/fcvm.2026.1862519 (PMC13549996; doi:10.3389/fcvm.2026.1862519)
Supplement: Supplementary file 1 [file Table1.docx]

**Supplementary Table 1. Variables used for clustering analysis**

| **Variable** | **Definition** | **Source variable** | **Unit** | **Type** | **Coding** | **Missing n (%)** | **Preprocessing** |
| --- | --- | --- | --- | --- | --- | --- | --- |
| AGE | Age at index hospitalization | BIRTH_DATE-derived | years | Continuous | Numeric | 0 (0.0%) | Derived before clustering; standardized to z-score |
| SEX | Biological sex | SEX | — | Binary categorical | Female=0; Male=1 | 0 (0.0%) | Binary encoding |
| ADM_SBP | Systolic blood pressure at admission | ADM_SBP | mmHg | Continuous | Numeric | 23 (0.3%) | Missing values imputed before clustering; standardized to z-score |
| ADM_HR | Heart rate at admission | ADM_HR | beats/min | Continuous | Numeric | 23 (0.3%) | Missing values imputed before clustering; standardized to z-score |
| ADM_HB | Hemoglobin at admission | ADM_HB | g/dL | Continuous | Numeric | 121 (1.6%) | Missing values imputed before clustering; standardized to z-score |
| ADM_CR | Serum creatinine at admission | ADM_CR | mg/dL | Continuous | Numeric | 123 (1.7%) | Missing values imputed before clustering; standardized to z-score |
| ADM_NA | Serum sodium at admission | ADM_NA | mmol/L | Continuous | Numeric | 129 (1.8%) | Missing values imputed before clustering; standardized to z-score |
| DISC_ECHO_LVEF_SIMPS | Left ventricular ejection fraction measured by Simpson's method | DISC_ECHO_LVEF_SIMPS | % | Continuous | Numeric | 1,315 (17.9%) | Missing values imputed before clustering; standardized to z-score |
| LOG_NTPROBNP | Log-transformed NT-proBNP at admission | ADM_NTPROBNP | log scale | Continuous | Natural log-transformed value | 1,796 (24.4%) | NT-proBNP was log-transformed; missing values imputed before clustering; standardized to z-score |
| ADM_CXR_PULCONG_YN | Pulmonary congestion on chest radiography at admission | ADM_CXR_PULCONG_YN | — | Binary categorical | No=0; Yes=1 | 169 (2.3%) | Missing values imputed before clustering; binary encoding |
| ADM_CXR_PLEUEFFU_YN | Pleural effusion on chest radiography at admission | ADM_CXR_PLEUEFFU_YN | — | Binary categorical | No=0; Yes=1 | 169 (2.3%) | Missing values imputed before clustering; binary encoding |
| ADM_CXR_CARDIOMEGALY_YN | Cardiomegaly on chest radiography at admission | ADM_CXR_CARDIOMEGALY_YN | — | Binary categorical | No=0; Yes=1 | 169 (2.3%) | Missing values imputed before clustering; binary encoding |
| DISC_HTN_YN | History of hypertension | DISC_HTN_YN | — | Binary categorical | No=0; Yes=1 | 90 (1.2%) | Missing values imputed before clustering; binary encoding |
| DISC_DM_YN | History of diabetes mellitus | DISC_DM_YN | — | Binary categorical | No=0; Yes=1 | 90 (1.2%) | Missing values imputed before clustering; binary encoding |
| DISC_CKD_YN | History of chronic kidney disease | DISC_CKD_YN | — | Binary categorical | No=0; Yes=1 | 92 (1.3%) | Missing values imputed before clustering; binary encoding |
| DISC_IHD_YN | History of ischemic heart disease | DISC_IHD_YN | — | Binary categorical | No=0; Yes=1 | 91 (1.2%) | Missing values imputed before clustering; binary encoding |
| DISC_COPD_YN | History of chronic obstructive pulmonary disease | DISC_COPD_YN | — | Binary categorical | No=0; Yes=1 | 91 (1.2%) | Missing values imputed before clustering; binary encoding |

Values in the Missing n (%) column indicate missingness in the source registry variables before preprocessing. Missing values were imputed before clustering. Continuous variables were standardized to z-scores, whereas categorical variables were encoded as binary indicators.

Abbreviations: ADM, admission; CKD, chronic kidney disease; COPD, chronic obstructive pulmonary disease; CR, creatinine; CXR, chest radiography; DISC, discharge; DM, diabetes mellitus; ECHO, echocardiography; HB, hemoglobin; HR, heart rate; HTN, hypertension; IHD, ischemic heart disease; LVEF, left ventricular ejection fraction; NA, sodium; NT-proBNP, N-terminal pro-B-type natriuretic peptide; PLEUEFFU, pleural effusion; PULCONG, pulmonary congestion; SBP, systolic blood pressure.
