# Supplementary material for: Distinct clinical phenotypes in acute heart failure identified by unsupervised clustering: a nationwide registry-based study
Source: Front Cardiovasc Med. 2026 Aug 25;13:1862519. doi: 10.3389/fcvm.2026.1862519 (PMC13549996; doi:10.3389/fcvm.2026.1862519)
Supplement: Supplementary file 2 [file Table2.docx]

**Supplementary Table 2. Cross-tabulation of cluster membership between the primary k-means clustering and Ward's hierarchical clustering**

| Primary k-means cluster | Ward Cluster 1 | Ward Cluster 2 | Ward Cluster 3 | Ward Cluster 4 | Ward Cluster 5 |
| --- | --- | --- | --- | --- | --- |
| Cluster 1 | 231 | 24 | 134 | 1,353 | 0 |
| Cluster 2 | 8 | 1,000 | 0 | 13 | 2 |
| Cluster 3 | 0 | 2 | 0 | 0 | 561 |
| Cluster 4 | 731 | 52 | 406 | 399 | 0 |
| Cluster 5 | 1,405 | 17 | 651 | 362 | 0 |

Data are presented as the number of patients assigned to each cluster. Ward's hierarchical clustering was performed using Ward's minimum variance method (Ward.D2) based on the same standardized variables used for the primary k-means clustering. The comparison demonstrates broad agreement between the two clustering approaches, with particularly high concordance for the original Cluster 2 and Cluster 3 phenotypes.
