# Supplementary material for: Distinct clinical phenotypes in acute heart failure identified by unsupervised clustering: a nationwide registry-based study
Source: Front Cardiovasc Med. 2026 Aug 25;13:1862519. doi: 10.3389/fcvm.2026.1862519 (PMC13549996; doi:10.3389/fcvm.2026.1862519)
Supplement: Supplementary file 3 [file Table3.docx]

Supplementary Table 3. Comparison of baseline characteristics between patients with available and missing left ventricular ejection fraction and NT-proBNP measurements before multiple imputation

| Characteristic | LVEF available (n=6036) | LVEF missing (n=1315) | P value | NT-proBNP available (n=5555) | NT-proBNP missing (n=1796) | P value |
| --- | --- | --- | --- | --- | --- | --- |
| Age, years | 68.8 ± 14.9 | 70.7 ± 14.6 | <0.001 | 68.8 ± 14.9 | 70.2 ± 14.6 | <0.001 |
| Male sex, n (%) | 3496 (57.9) | 675 (51.3) | <0.001 | 3198 (57.6) | 973 (54.2) | 0.013 |
| SBP at admission, mmHg | 134.6 ± 28.7 | 132.6 ± 28.4 | 0.021 | 133.2 ± 28.1 | 137.7 ± 30.2 | <0.001 |
| Heart rate at admission, bpm | 91.8 ± 24.9 | 88.1 ± 25.3 | <0.001 | 90.5 ± 24.8 | 93.2 ± 25.6 | <0.001 |
| Hypertension, n (%) | 3519 (58.9) | 810 (62.9) | 0.009 | 3318 (59.8) | 1011 (59.0) | 0.536 |
| Diabetes mellitus, n (%) | 2396 (40.1) | 508 (39.4) | 0.678 | 2219 (40.0) | 685 (39.9) | 0.982 |
| Chronic kidney disease, n (%) | 967 (16.2) | 225 (17.5) | 0.269 | 833 (15.0) | 359 (20.9) | <0.001 |
| Ischemic heart disease, n (%) | 1672 (28.0) | 311 (24.2) | 0.006 | 1429 (25.8) | 554 (32.3) | <0.001 |
| COPD, n (%) | 459 (7.7) | 112 (8.7) | 0.241 | 378 (6.8) | 193 (11.3) | <0.001 |

Values are presented as mean ± standard deviation or n (%), as appropriate. Comparisons were performed using Welch's t-test for continuous variables and the chi-square test or Fisher's exact test for categorical variables, as appropriate. Missingness refers to the absence of the respective LVEF or NT-proBNP measurement in the original registry dataset before imputation.
